# Silencing of *Eag1* Gene Inhibits Osteosarcoma Proliferation and Migration by Targeting STAT3-VEGF Pathway

**DOI:** 10.1155/2015/617316

**Published:** 2015-12-09

**Authors:** Xinyu Wu, Zhida Chen, Wengrong Zeng, Yuanfu Zhong, Qingjun Liu, Jin Wu

**Affiliations:** ^1^Department of Neurology, The Affiliated Southeast Hospital of Xiamen University, Zhangzhou 363000, China; ^2^Department of Orthopaedics, The Affiliated Southeast Hospital of Xiamen University, Orthopaedic Center of People's Liberation Army, Zhangzhou 363000, China; ^3^Central Laboratory, The Affiliated Southeast Hospital of Xiamen University, Zhangzhou 363000, China

## Abstract

So far, the role of Ether à go-go 1 (Eag1) potassium channels in migration and invasion progression of cancers remains elusive. In the present study, the effects of* Eag1* knockdown on osteosarcoma cell proliferation, growth, and apoptosis were examined. Then, we evaluated the effects of* Eag1* silencing on osteosarcoma cell migration and invasion. In addition, we detected the expression of vascular endothelial growth factor (VEGF) and signal transducer and activator of transcription 3 (STAT3) in osteosarcoma cell treated with Eag1 small interfering RNAs (siRNAs). Finally, STAT3 siRNA was employed to determine the influence of downregulation of STAT3 on cell proliferation and migration. The results showed that knockdown of* Eag1* significantly suppressed osteosarcoma cell proliferation and osteosarcoma xenografts growth. However,* Eag1* silencing had little effect on cell apoptosis. Additionally, osteosarcoma cell adhesion, migration, and invasion were also potently attenuated. Notably, the expression levels of VEGF decreased evidently upon Eag1 siRNAs treatment, paralleled with reductions in the expression levels of STAT3. Moreover, a similar pattern was observed in osteosarcoma cell proliferation and migration suppression between STAT3 siRNA and Eag1 siRNAs groups. Our data indicated that Eag1 promotes osteosarcoma proliferation and migration, at least in part, by targeting STAT3-VEGF pathway.

## 1. Introduction

Osteosarcoma (OS) is the most common primary malignant bone tumor in the adolescent age group, with a second peak incidence in geriatric patient populations [1, 2]. OS cells are originated in osteoblast committed cells [3] and characterized by proliferous tumor cells to form immature bone or bone tissue. It is a very aggressive cancer which if left untreated is universally fatal [4, 5]. With the rapid development of treatment for high grade OS by the combination of surgery with neoadjuvant chemotherapy, 5-year survival rates of patients presenting without metastatic disease have reached 60–75% [6]. However, 40–50% of patients will develop metastases that are difficult to treat and confer a poor prognosis [7]. Meanwhile, high-dose chemotherapy has lots of adverse reactions which restrict its application. Therefore, development of novel treatment strategies is critical for the improvement of the prognosis of OS patients. Recently, a profound genetic instability leading to the aberrant and uncoordinated expression of several gene products has been found to be associated with OS, which may represent potential targets for osteosarcoma diagnosis and treatment. One such potential target is the voltage-gated potassium (Kv) channels.

In recent years, the functional role of Kv channels in cancer biology has been an area of intense investigation [8]. Several Kv channels especially Eag1 (Kv10.1, KCNH1) channels have shown close relation to cancer growth, progression, and metastasis. The* EAG* gene was originally cloned from* Drosophila melanogaster* in 1969 [9] and formed by three subfamilies: Eag, Erg (the eag-related gene), and Elk (the eag-like gene). Two members of the Eag subfamily are Eag1 and Eag2 (Kv10.2, KCNH5), respectively [10]. Interestingly, the physiological expression of Eag1 is largely restricted to the brain; however Eag1 is ectopically expressed in several tumors [11, 12]. In fact, this restricted distribution in normal tissues is one of the most attractive features of Eag1 as a potential tumor marker. Moreover, numerous* in vitro* and* in vivo* studies have strongly suggested the involvement of Eag1 in cancer progress and its oncogenic potential [13–16].

Although our previous study has demonstrated the aberrant expression and possible regulation mechanism of Eag1 in OS [17, 18], it is unclear whether Eag1 is implicated in migration and invasion of OS. Migration and invasion are an important feature of OS and their therapeutic inhibition might be critical to avoid metastasis of OS. Unfortunately, only few studies have focused on the relationship between Eag1 and cancer migration and invasion. In 2010, the effects of Eag1 inhibitors on human myeloid leukemia cell lines migration were detected and the results indicated an implication of Eag1 in this process [19]. Later on, studies demonstrated that Eag1 is involved in the serum-induced migration of breast cancer cells by controlling the Ca^2+^ entry [20].

In this study, we performed* in vitro and in vivo* experiments to evaluate the effects of Eag1 knockdown on proliferation, apoptosis, migration, and invasion of MG-63 and Saos-2 cells. We also examined the underlying mechanisms by which the inhibition of OS cell proliferation and migration is induced by specific blockade of Eag1.

## 2. Materials and Methods

### 2.1. Cell Culture and Transfection

Human OS cell lines MG-63 and Saos-2 were purchased from the American type culture of collection (ATCC). The cells were, respectively, cultured at 37°C, in a humidified atmosphere in 5% CO_2_ and 95% air in RPMI-1640 medium (Gibco, Rockville, MD, USA) supplemented with 10% fetal bovine serum (FBS; Gibco), 100 U/mL penicillin, and 100 *μ*g/mL streptomycin, and subcultured every 3-4 days.

27 mer siRNA duplexes for human Eag1 (ID 3756), STAT3 (ID6774), and trilencer-27 universal scrambled negative control siRNA duplex (SR30004) were obtained from OriGene (Rockville, MD, USA). Cells at 70% confluence were transfected with 27 mer siRNA duplexes for human Eag1, STAT3, and trilencer-27 universal scrambled negative control siRNA duplex by using Lipofectamine RNAiMAX (Invitrogen, Rockville, MD, USA) according to the manufacturer's protocol.

### 2.2. Real-Time PCR

The total RNA was isolated from the cultured cells or tumor tissues of nude mice by Trizol reagent (Invitrogen, Rockville, MD, USA). Real-time PCR was carried out using LightCycler 480 Probes Master Kit (Roche Diagnostics, Mannheim, Germany), according to the following protocol: DNA denaturation at 95°C for 10 min, followed by 45 amplification cycles consisting of 10 s at 95°C, 30 s at 60°C, and 1 s at 72°C. Primers sequences were designed as follows: for Eag1: forward primer 5′-GCT TTT GAG AAC GTG GAT GAG-3′; backward primer 5′-CGA AGA TGG TGG CAT AGA GAA-3′. For *β*-actin: forward primer 5′-TCC ACC TTC CAG CAG ATG TG-3′; backward primer 5′-GCA TTT GCG GTG GAC GAT-3′.

### 2.3. Western Blot Analysis

5-6 × 10^7^ cells were washed twice with Phosphate-Buffered Saline (PBS) before being collected and lysed in lysis buffer (50 mmol/L Tris-Cl (pH 7.5), 150 mmol/L NaCl, 0.2 mmol/L EDTA, 1 mmol/L PMSF, and 1% (v/v) Nonidet-P40) for 30 min. The crude lysate was obtained by centrifugation at 13,200 rpm for 10 min at 4°C. 25 *μ*g protein samples were separated by a 12% sodium dodecyl sulfate-polyacrylamide gel electrophoresis (SDS-PAGE) and transferred to nitrocellulose (Bio-Rad, Richmond, CA). After being blocked in 10% (w/v) nonfat milk powder (Sigma, St. Louis, MO, USA) at room temperature for 1 h, the membranes were incubated with antibodies against Eag1, VEGF, STAT3 (Abcam, Cambridge, MA), and GAPDH (Santa Cruz Biotechnology, CA, USA) overnight. Secondary antibodies were chosen according to the species of origin of the primary antibodies (Santa Cruz). Then the membranes were developed with chemiluminescent detection kit (Zhongshan Biotechnology, Beijing, China) and exposed to X-ray films.

### 2.4. Cell Proliferation Assay

The cell proliferation was analyzed by using Cell Counting Assay Kit-8 (CCK-8; Dojindo Molecular Technologies, Gaithersburg, MD) according to the manufacturer's protocol. In brief, 1 × 10^5^ cells were starved in serum-free medium for 12 h and then the cells were transfected. After 48 h, cells were harvested. Ten microliters of Cell Counting Assay Kit-8 solution was added to each well, the cells were incubated for another 1 h, and the absorbance (*A*) at 490 nm was measured by using spectrophotometer (Bio-Rad, Richmond, CA, USA). Experiments were performed at least three times with representative data presented.

### 2.5. Colony Formation Assay

For the colony formation assay, 0.5% agar (Sigma) was added to 60 mm dishes. The treated OS cells were mixed with 0.3% soft agar and placed on the bottom agar with 1 × 10^3^ cells per dish. The cells were incubated for up to 2 weeks till the colonies were clearly visible even without looking under the confocal microscope (Olympus, Japan).

### 2.6. Xenograft Experiments

Thymus-null BALB/c nude mice (female, age 4–6 weeks) were obtained from the Animal Center of Chinese Academy of Medical Sciences. All animal procedures were performed according to approved protocols and in accordance with recommendations for the proper use and care of laboratory animals. Nude mice were inoculated subcutaneously with 0.5 mL (1 × 10^5^ cells/mL) of transfected OS cells and the size of the tumor was measured every 5 days. The animals were killed 25 days later. Tumor growth was measured using caliper and tumor volume (cm^3^) was determined with the following formula: *ab* ^2^/2, where *a* was the length and *b* was the width of the tumor.

### 2.7. Flow Cytometry Analysis

Apoptotic rate was determined by flow cytometry analysis using an Annexin V-FITC Apoptosis Kit. The cells were collected and washed twice with cold PBS 48 h after the transfection with siRNA and then resuspended at 1 × 10^6^ cells/mL and fixed in 70% cold ethanol overnight at 4°C. Staining was performed according to the manufacturer's instructions, and flow cytometry was performed on a FACScan flow cytometer (Becton Dickinson, San Jose, CA). The percentage of the early apoptosis was calculated by counting cells positive for annexin V and negative for propidium iodide (PI), while the percentage of the late apoptosis was calculated by counting cells positive for both annexin V and PI.

### 2.8. Cell Adhesion Assay

1 × 10^5^ cells were plated on collagen I (5 *μ*g/cm^2^) coated 96-well plates (Sigma) for 24 h. After transfection with different siRNAs for 4 h, cells were washed with PBS and fixed with 70% ethanol and then stained with crystal violet (0.1% in 20% methanol) for 10 min. After washing with water, the wells were allowed to dry. The crystal violet was dissolved in 10% acetic acid, and the absorbance was measured at 490 nm.

### 2.9. Wound Healing Assay

Cells at a density of 5 × 10^5^ cells/well were cultured in 6-well plates (Sigma) with serum-free RPMI-1640 medium until they were 100% confluent in an adherent monolayer. A sterile yellow 10 *μ*L Eppendorf tip (Sigma) was used to scratch the cells in the plate and then washed with PBS three times. The cells were then placed in fresh serum-free RPMI-1640 medium for 24 h; then random fields were examined, selected, and photographed with an inverted microscope (Olympus).

### 2.10. Cell Invasion Assay

Cell invasion assay was performed in 24-well matrigel-coated Transwell chambers (8 *μ*m pore size, Corning, NY, USA) according to the manufacturer's instructions. Cells were collected 24 h after transfection with siRNAs and 2 × 10^4^ cells in 0.1% FBS medium were seeded per upper chamber, while 5% FBS medium was placed in the bottom chamber. After 24 h, the chambers were removed; the invasive cells were stained with Giemsa and photographed under the microscope. The number of invading cells was determined by counting ten high-power fields (×400) on each membrane and calculated as the mean number of cells per field.

### 2.11. Statistical Analysis

All data were presented as mean ± standard error of mean (SEM). Statistical significance was determined using *t*-test or analysis of variance (ANOVA) using the SPSS18.0 program. *P* < 0.05 was considered as significant difference.

## 3. Results

### 3.1. Eag1 Knockdown Inhibits OS Cell Proliferation* In Vitro*

**Figure 1:**
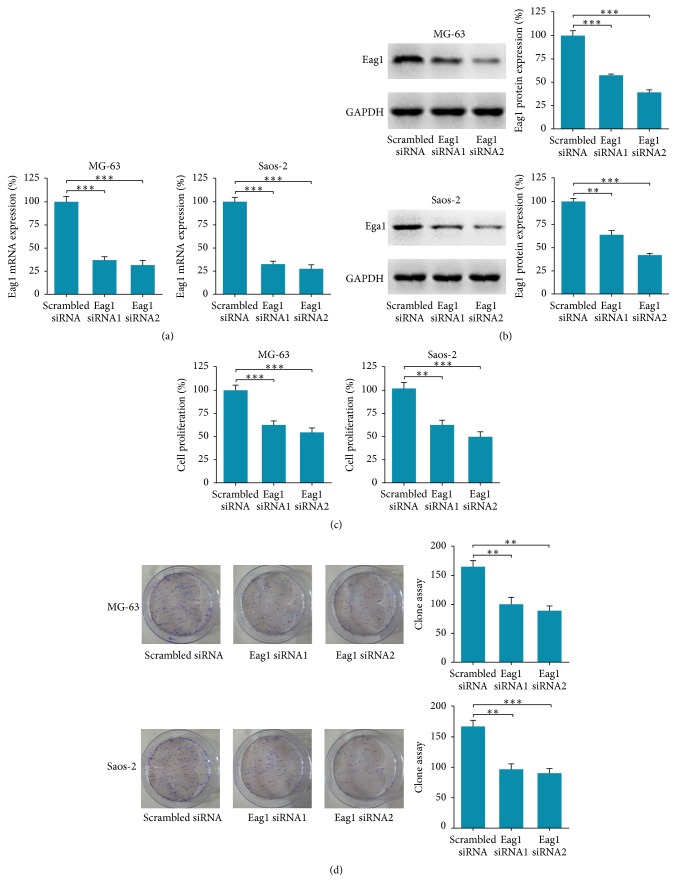
Eag1 knockdown reduces proliferation and growth of OS cells. (a) and (b) Efficiency of Eag1 knockdown by Eag1 siRNAs was measured by RT-PCP and Western blot (*n* = 3). (c) The proliferation of MG-63 and Saos-2 cells was determined by CCK-8 assay after transfection with Eag1 siRNAs. Data were presented as mean ± SEM (*n* = 3). (d) The growth of MG-63 and Saos-2 cells was determined by colony formation assay (*n* = 3). ^*∗∗*^ *P* < 0.01, ^*∗∗∗*^ *P* < 0.001.

To characterize the possible role of Eag1 in OS cells, we first knocked down Eag1 expression in OS cells by transfecting with Eag1 siRNAs. Compared to scrambled siRNA transfected cells, the cells transfected with Eag1 siRNAs had significantly reduced Eag1 mRNA (Figure 1(a)) and protein (Figure 1(b)) expression levels, indicating that Eag1 siRNAs had a high knockdown efficiency. Then, we checked the cell proliferation and growth using CCK-8 assay (Figure 1(c)) and colony formation assay (Figure 1(d)) following the transfection of MG-63 and Saos-2 cells with Eag1 siRNA1 and siRNA2, respectively. The results demonstrated that Eag1 siRNAs inhibited the proliferation and colony formation ability of MG-63 and Saos-2 cells effectively, suggesting that Eag1 functions as a tumor promoter in OS cells.

### 3.2. Eag1 Knockdown Inhibits Tumor Growth in Xenograft Model of OS

**Figure 2:**
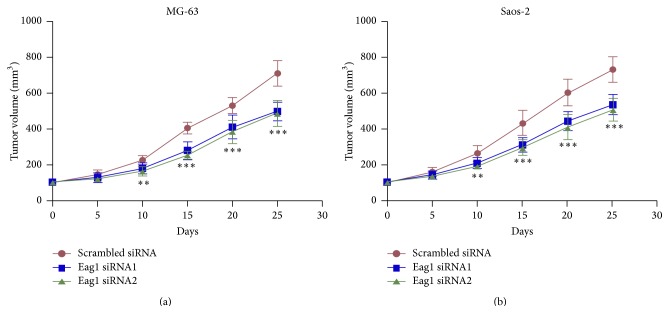
Eag1 siRNAs inhibit OS growth* in vivo*. MG-63 and Saos-2 cells transfected with indicated siRNAs were xenografts in nude mice. The length and width of tumor were measured weekly after inoculation and the volume of tumor was calculated. After 25 days, the tumor volume growth curve was drafted (*n* = 6). ^*∗∗*^ *P* < 0.01, ^*∗∗∗*^ *P* < 0.001.

To extend our* in vitro* observation on cultured OS cells, we made a xenograft model of OS using cells transfected with scrambled siRNA, Eag1 siRNA1, or Eag1 siRNA2. The tumor volumes of animals in scrambled siRNA group were significantly smaller than that in Eag1 siRNA1 or Eag1 siRNA2 group (Figure 2). These* in vivo* data confirmed that Eag1 promotes OS growth.

### 3.3. The Effect of Eag1 Knockdown on OS Cell Apoptosis

**Figure 3:**
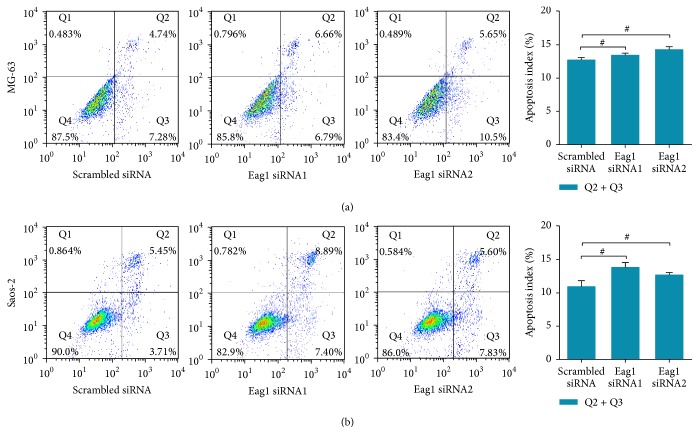
The influence of Eag1 siRNA on the apoptosis of OS cells. There was no statistical difference in the total apoptotic ratio (Q2 + Q3) between MG-63 and Saos-2 cells transfected with scrambled siRNA or Eag1 siRNA. ^#^ *P* > 0.05, *n* = 3. Q2 quadrant (annexin V+, PI+) represented late apoptotic cells and Q3 quadrant (annexin V+, PI−) represented early apoptotic cells.

The results of flow cytometry showed that there was no statistically significant difference between OS cells transfected with scrambled siRNA or Eag1 siRNAs (Figure 3).

### 3.4. Eag1 Knockdown Inhibits Adhesion, Migration, and Invasion of OS Cells

**Figure 4:**
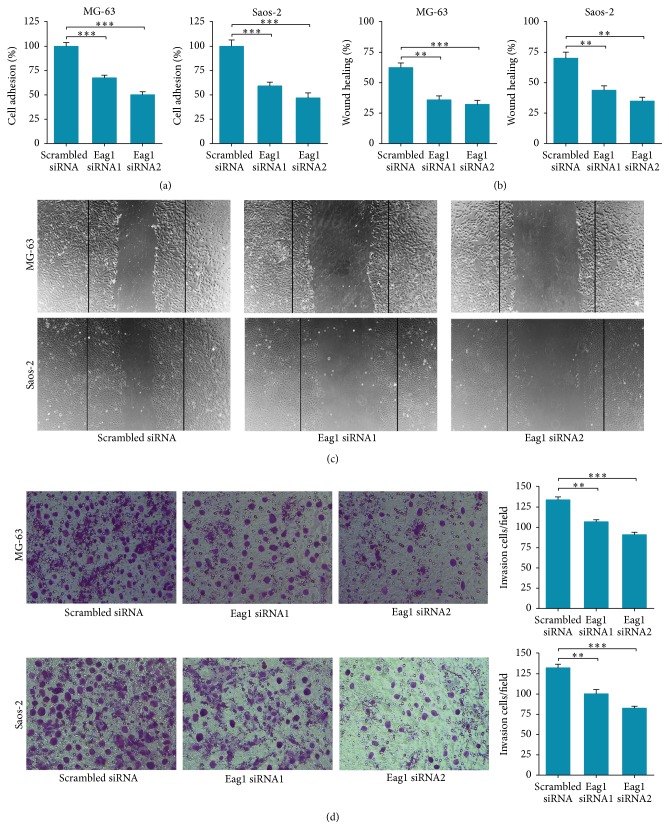
Eag1 knockdown suppresses adhesion, migration, and invasion of OS cells. (a) MG-63 and Saos-2 cells were transfected with indicated siRNAs and cell adhesion ability was evaluated by adhesion assay as described in Section 2 (*n* = 3, the results from scrambled siRNA group were given as 1.0). (b) MG-63 and Saos-2 cells transfection with scrambled siRNA or Eag1 siRNAs were wounded and then in fresh serum-free RPMI-1640 medium. After 24 h, migrating cells in the wound were counted (*n* = 3). (c) Representative pictures of the wounds at random locations. ^*∗∗∗*^ *P* < 0.001. (d) Representative microscopic images of the Transwell insert of transfected OS cells. MG-63 and Saos-2 cells were grown on Transwell inserts coated with collagen I and after transfection with scrambled siRNA or Eag1 siRNAs, invaded cells were counted. Each bar gives the mean ± SEM of 3 experiments. ^*∗∗*^ *P* < 0.01.

To understand the role of Eag1 in regulation of cancer migration and invasion, we assessed the effects of Eag1 knockdown on OS cell by performing adhesion assay, wound healing assay, and Transwell invasion assay. The results showed that MG-63 and Saos-2 cells transfected with Eag1 siRNAs exhibited reduced cell adhesion (Figure 4(a)), migration (Figures 4(b) and 4(c)), and invasion (Figure 4(d)) compared with the cells transfected with scrambled siRNA.

### 3.5. Eag1 Regulates STAT3-VEGF Pathway in OS Cells

**Figure 5:**
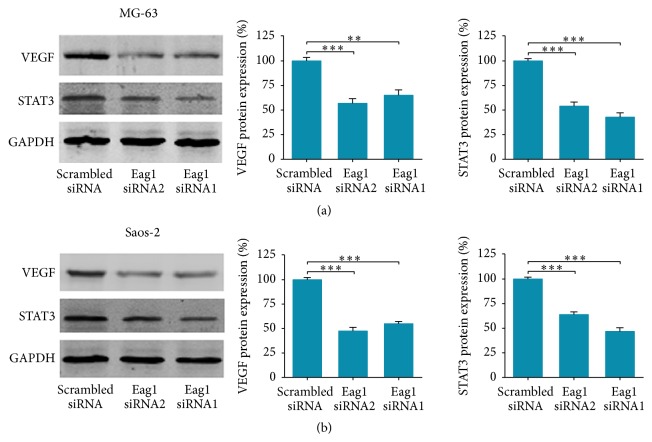
Eag1 knockdown downregulates STAT3-VEGF expression* in vitro*. (a) 27 mer siRNA duplexes for human Eag1 or trilencer-27 universal scrambled negative control siRNA duplex was transfected into MG-63 cells. Two days later the cells were harvested and subjected to Western blot using VEGF and STAT3 antibodies. Transfection with Eag1 siRNAs resulted in reduced VEGF and STAT3 expression. (b) Similar results were observed in Saos-2 cells transfected with Eag1 siRNAs. *n* = 3, ^*∗∗*^ *P* < 0.01 versus scrambled siRNA group.

VEGF expression is upregulated in many human cancers and plays an important role in cancer growth, invasion, and angiogenesis [21]. Given the oncogenic role of Eag1 in OS we showed above, we postulated that VEGF may mediate pro-proliferative and migration effects of Eag1 in OS. We detected the expression of VEGF in MG-63 and Saos-2 cells transfected with Eag1 siRNAs or scrambled siRNA. Western blot analysis showed that transfection with Eag1 siRNAs resulted in a significant reduction of VEGF expression (Figures 5(a) and 5(b)). This result suggested that Eag1 may upregulate the expression of VEGF in OS cells, consistent with the results that cancer cells express Eag1 show significantly higher levels of VEGF secretion than controls [22].

**Figure 6:**
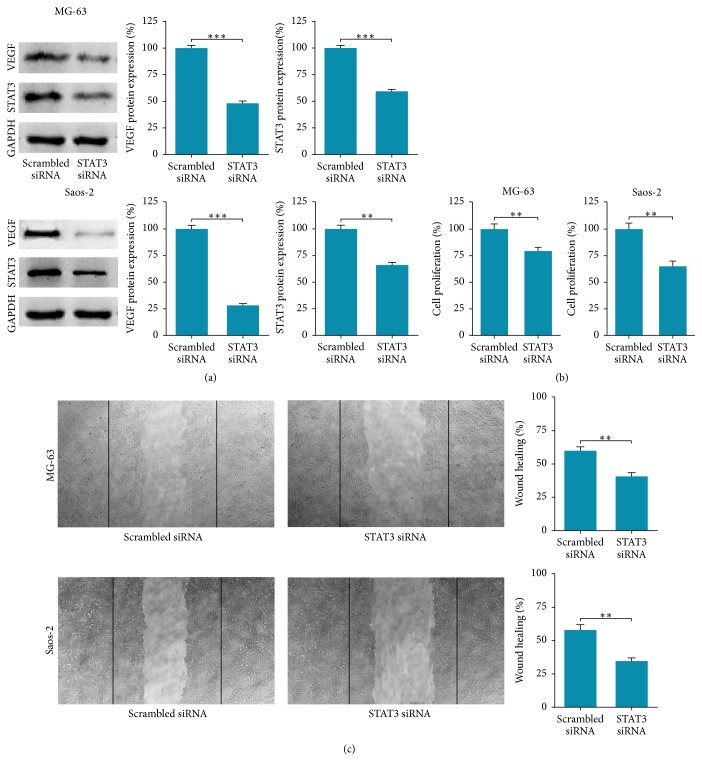
The inhibition of proliferation and migration mediated by STAT3 siRNA. (a) Endogenous STAT3 and VEGF expression was efficiently repressed by STAT3 siRNA in MG-63 and Saos-2 cells. (b) Inhibition of cell proliferation via STAT3 knockdown. (c) The effects of STAT3 knockdown on the migration of OS cells. ^*∗∗*^ *P* < 0.01, ^*∗∗∗*^ *P* < 0.001.

To further explore the exact mechanism by which Eag1 regulates the VEGF expression levels, we focused on STAT3 which contributes to cancer development and progression in numerous forms of cancers including OS [23]. Moreover, STAT3 protein binds to the VEGF promoter and regulates VEGF directly [24]. Thus, the effects of Eag1 knockdown on STAT3 expression were investigated. When the MG-63 and Saos-2 cells were transfected with Eag1 siRNAs for the indicated times, the expression levels of STAT3 decreased significantly, paralleled with the reduction in the VEGF expression levels (Figures 5(a) and 5(b)). In addition, to further evaluate whether Eag1 suppresses cell proliferation and migration by downregulating STAT3-VEGF pathway, cells were transfected with STAT3 siRNA. As shown in Figure 6(a), similar reductions in STAT3 and VEGF protein were induced by STAT3 siRNA in MG-63 and Saos-2 cells. Additionally, silencing of STAT3 significantly suppressed the proliferation (Figure 6(b)) and migration (Figure 6(c)) of OS cell. Collectively, these results demonstrated that the pro-proliferation and promigration effects of Eag1 may be via STAT3-VEGF pathway.

## 4. Discussion

To inhibit the invasion and metastasis of OS and reduce associated side effects of conventional chemotherapeutic drugs, new therapy strategies are necessary. We focused on Eag1 channel because of its restricted distribution in normal tissue and more ubiquitous distribution in cancer cells and its oncogenic properties [25]. Some chemotherapeutic drugs such as imipramine and astemizole have been shown to inhibit Eag1 and decrease cell proliferation in cancer cell lines [14]. Unfortunately, there are no specific blockers for Eag1 [11]. Additionally, both imipramine and astemizole have undesirable cardiovascular side effects which limit their applicability in treating cancer [16].

RNA interference (RNAi) has become a simple and effective genetic tool to specifically silence gene expression. In contrast to chemotherapeutic drugs, Eag1 RNAi has been shown to inhibit the proliferation of various cancer cell lines with minimal nonspecific side effects [13]. Thus, the use of Eag1 siRNA may be a potential new approach to the treatment of cancers including OS. Although Eag1 has extensively been studied concerning its implication in cancer cell proliferation and tumorigenesis, so far little attention has been drawn to its role in cancer migration and invasion. As we know, OS is characterized by a highly malignant tendency to invade the surrounding tissues and to metastasize. Furthermore, pulmonary metastasis is the main cause of death in patients with OS. Hence, it is important to find out the role and molecular mechanisms of Eag1 in the migration and invasion of OS and the study results may provide for a new targeted therapeutic agent in OS treatment.

To establish a useful model for further studies, we tested the role of Eag1 in two well-established OS cell lines MG-63 and Saos-2. We observed significant reduction of proliferation and colony formation in two cell lines after treatment with Eag1 siRNAs, in agreement with previous study [13]. Interestingly, it is well known that silencing by siRNA generally lasts for a week at the longest. We indeed detected protein knockdown level at least starting from 48 h after siRNA transfection and showed inhibitory effects on colony formation assay (2 weeks) and xenograft models (25 days). Together, our data here indicates that the deficiency of Eag1 between 48 h and 7 days is sufficient to bring about cell proliferation deficit that still sustains to 2 weeks or even longer time. Next, to explore the mechanism by which Eag1 promotes the growth of OS cells, we examined apoptosis following Eag1 knockdown by double staining with annexin V and PI. The results demonstrated that* Eag1* silencing had little effect on cell apoptosis. Possibly, Eag1 siRNA exerts its antiproliferative effect through cell-cycle-specific rather than apoptotic mechanisms [26]. Then, we focused on the role of Eag1 in the migration and invasion of OS. The results demonstrated that Eag1 siRNAs lead to reduced adhesion, migration, and invasion of MG-63 and Saos-2 cells. Finally, we tried to explain the detailed mechanisms by which OS cell adhesion, migration, and invasion are inhibited by specific blockade of Eag1. Among the candidate target genes, we focused on VEGF because of its known role as a regulator of critical functions in cancer migration and invasion [27]. Moreover, Eag1 expression increases basal expression levels of hypoxia-inducible factor 1*α* (HIF1*α*), resulting in the upregulation of VEGF [22]. So we examined the expression level of VEGF using Western blot analysis and the results showed that Eag1 knockdown leads to reduced expression of VEGF in MG-63 and Saos-2 cells.

Because of the important roles in cancer survival, proliferation, apoptosis, and metastasis, STAT proteins have gained interest as promising tools for the development of novel therapeutic methods of cancers [28, 29]. Among the STAT proteins, STAT3 stands out as the most attractive one in relation to its contributions to development and progression in numerous forms of cancer, including OS [23]. Accumulating evidence suggests that STAT3 is one of the major transcription activators of VEGF [30] and contributes to cancer invasion and metastasis via regulation expression of VEGF [31]. So next we investigated the effect of Eag1 knockdown on STAT3 expression by performing Western blot analysis. Our study showed that the expression levels of STAT3 decreased significantly, paralleled with the reduction in the VEGF expression levels in OS cells transfection with Eag1 siRNAs. Furthermore, to determine whether Eag1 siRNAs inhibit cell migration and invasion by downregulating STAT3, OS cells were transfected with STAT3 siRNA. Similar reductions in proliferation and migration were induced by STAT3 siRNA in OS cells. These data suggested that Eag1 may promote OS proliferation and migration, at least in part, through positive regulation of STAT3-VEGF pathway.

Taken together, by using loss-of-function approach, our study provides* in vitro* and* in vivo* evidence for the role of Eag1 in promoting the proliferation, adhesion, migration, and invasion of OS cells. Further mechanistic studies to understand the role of Eag1 in the regulation of adhesion, migration, and invasion of OS cells will help develop Eag1 siRNA as a novel therapeutic approach to treatment of OS and improve the prognosis of OS. However, we do note that this study has certain limitations. Our results showed that Eag1 siRNA can inhibit OS cells adhesion, migration, and invasion through the STAT3/VEGF pathway. However, the regulation of migration and invasion-related cytokines in OS cells is quite complex. We do not rule out the detailed mechanisms of how Eag1 regulates the STAT3-VEGF pathway or the possibility that other pathways that modulate VEGF expression may also be affected by Eag1 siRNA. Moreover, we also do not rule out the molecular relationship or molecular mechanisms between Eag1 and STAT3. Previous study has demonstrated the role of IL-6R/STAT3/miR-34a feedback loop in colorectal cancer invasion and metastasis [32], and our previous study has shown the relationship between miRNA-34a and Eag1 [18]. Thus, we assume the connection between miRNA-34a, STAT3, and Eag1 and all of these will be our next aim.
